# Supplementary material for: Clinical Outcomes of 3D-Printed Titanium Patient-Specific Implants in Lumbar Interbody Fusion: A Prospective Clinical Trial with a Systematic Review of Conventional Techniques
Source: J Pers Med. 2025 Jul 16;15(7):320. doi: 10.3390/jpm15070320 (PMC12300856; doi:10.3390/jpm15070320)
Supplement: Supplementary file 1 [file jpm-15-00320-s001.zip › jpm-3693051-supplementary.pdf]

Clinical Outcomes of 3D Printed Titanium Patient-Specific Implants in Lumbar Interbody Fusion; A Prospective Clinical Trial with a Systematic Review of Conventional Techniques

Appendix and Supplementary Information

Supplementary Method

QoL Information: The EQ-5D-5L can be used to determine QoL indices using a value set for a given population. We used the Australian EQ-5D-5L value set derived by Norman et al. 2023 to calculate QoL Index scores. For the Australian Spine Registry that used the EQ-5D-3L survey, we used the 'valuations of the 243 EQ-5D States' from Viney et al. 2011.

Table S1: Patient Demographics of Investigational (3DMorphic) cohort.

Patient ID	Age	Gender	Level Treated	Surgical Approach	Supplementary instrumentation?
1	36	M	L5/S1	ALIF	Yes - Disc replacement L4/5
2	35	F	L3/4, L4/5	LLIF	No
3	75	M	L4/5, L5/S1	ALIF	No
4	57	M	L5/S1	ALIF	Yes - Posterior rods and screws
5	64	F	L4/5, L5/S1	ALIF	Yes - Posterior rods and screws
6	79	M	L3/4, L4/5	ALIF	Yes - Posterior rods and screws
7	78	F	L1/2, L3/4	LLIF	No
8	39	M	L5/S1	ALIF	Yes - Posterior rods and screws
9	63	F	L5/S1	ALIF	Yes - Posterior rods and screws
10	78	M	L3/4, L4/5	LLIF	Yes - Posterior rods and screws
11	51	F	L4/5	LLIF	Yes - Posterior rods and screws
12	74	F	L5/S1	ALIF	Yes - Posterior rods and screws
13	62	F	L3/4	LLIF	Yes - Posterior rods and screws
14	37	M	L5/S1	ALIF	No
15	79	F	L4/5	LLIF	No
16	46	M	L5/S1	ALIF	Yes - Posterior rods and screws
17	56	M	L3/4, L4/5	LLIF	Yes - Posterior rods and screws
18	47	F	L5/S1	ALIF	Yes - Disc replacement L4/5
19	30	M	L5/S1	ALIF	Yes - Posterior rods and screws

20	55	M	L5/S1	ALIF	Yes - Disc replacement L3/4
21	66	F	L3/4	LLIF	Yes - Posterior rods and screws
22	67	M	L4/5, L5/S1	ALIF	No
23	76	M	L4/5	LLIF	Yes - Posterior rods and screws
24	55	M	L4/5, L5/S1	ALIF	Yes - Posterior rods and screws
25	33	M	L5/S1	ALIF	Yes - Posterior rods and screws
26	30	F	L5/S1	ALIF	No
27	50	M	L4/5, L5/S1	ALIF	Yes - Posterior rods and screws
28	79	M	L4/5	LLIF	Yes - Posterior rods and screws
29	36	F	L4/5	ALIF	No
30	68	M	L4/5, L5/S1	ALIF	Yes - Anterior Plate
31	74	M	L2/3	LLIF	Yes - Posterior rods and screws
32	71	F	L2/3	ALIF	Yes - Posterior rods and screws
33	77	F	L3/4	ALIF	Yes - Posterior rods and screws
34	49	M	L4/5	ALIF	No
35	65	M	L5/S1	ALIF	No
36	66	F	L4/5	LLIF	Yes - Posterior rods and screws
37	74	M	L2/3	LLIF	Yes - Posterior rods and screws
38	20	M	L5/S1	ALIF	Yes - Posterior rods and screws
39	58	M	L5/S1	ALIF	No
40	27	F	L4/5	ALIF	No
41	56	M	L5/S1	ALIF	Yes - TDR at L45
42	69	F	L3/4, L4/5, L5/S1	LLIF	Yes - Posterior rods and screws. Also, PLIF at L5/S1 level
43	73	F	L3/4, L4/5	LLIF	Yes - Posterior rods and screws
44	53	M	L4/5, L5/S1	ALIF	No
45	32	F	L5/S1	ALIF	No
46	72	F	L1/2, L5/S1	LLIF, ALIF	Yes - posterior rods and screws
47	24	M	L5/S1	ALIF	Yes - posterior rods and screws
48	68	F	L2/3, L4/5	LLIF	Yes - posterior rods and screws
49	51	F	L5/S1	ALIF	No
50	57	M	L3/4	LLIF	No
51	74	F	L4/5	LLIF	No
52	62	M	L5/S1	ALIF	No
53	76	M	L4/5, L5/S1	ALIF	Yes - posterior rods and screws
54	42	F	L5/S1	ALIF	No
55	50	F	L5/S1	ALIF	Yes - posterior rods and screws
56	66	M	T11/12	LLIF	Yes - posterior rods and screws
57	30	M	L3/4, L5/S1	LLIF, ALIF	Yes - posterior rods and screws
58	62	F	L4/5, L5/S1	ALIF	No
59	71	M	L5/S1	ALIF	No
60	58	M	L5/S1	ALIF	No
61	31	F	L5/S1	ALIF	Yes - TDR at L45
62	59	M	L3/4, L4/5, L5/S1	ALIF	No
63	77	F	L2/3, L3/4	LLIF	Yes - posterior rods and screws

64	52	F	L4/5	LLIF	Yes - posterior rods and screws
65	30	M	L4/5	LLIF	Yes - posterior rods and screws
66	87	M	L4/5	LLIF	No
67	74	M	L2/3, L3/4	LLIF	Yes - posterior rods and screws
68	78	F	L3/4, L4/5, L5/S1	LLIF, ALIF	Yes - posterior rods and screws
69	77	M	L3/4, L4/5, L5/S2	LLIF, ALIF	Yes - posterior rods and screws
70	51	M	L5/S1	ALIF	Yes - posterior rods and screws
71	71	F	L2/3	LLIF	No
72	38	M	L3/4, L4/5	ALIF	Yes - posterior rods and screws
73	57	F	L4/5, L5/S1	LLIF, ALIF	Yes - posterior rods and screws
74	64	M	L3/4, L5/S1	ALIF	Yes - posterior rods and screws
75	75	M	L2/3, L4/5	LLIF	Yes - posterior rods and screws
76	39	M	L5/S1	ALIF	Yes - posterior rods and screws
77	72	F	L4/5	LLIF	Yes - posterior rods and screws
78	41	F	L5/S1	ALIF	Yes – TDR at L45

Equation S1

$$Final\ Estimate = \sum\left(\frac{n_i}{n_{total}} \times Score_i\right)$$

Where n_i and $Score_i$ is the sample size and VAS score of the ith study, respectively, and n_{total} is the sum of all sample sizes across studies included in group analysis.

Supplementary Results

Table S2: Off-The-Shelf cage designs (cage reference numbers).

Cage Design	Reference Code
Non-Integral Screw Fixation Cages	NISF
Integral Screw Fixation Cages	ISF
Bagby and Kuslich Cages	BAK
Femoral Ring Allograft	FRA

Table S3: Cages used amongst RCT identified in the systematic review.

**CAGE INFO**	**CAGE REFERENCE**
SynCage	NISF-SC
Femoral Ring Allograft	FRA
4WEB Anterior Spine Truss System	NISF-4W
BAK fusion cage	BAK
SynFix LR	ISF-SF
XLIF (Nuvasive)	NISF-XLIF
Carbon fibre reinforced polymer ALIF cage (DePuy Synthes Inc.)	NISF-CF
